# Imaging tests in staging and surveillance of non-metastatic breast cancer: changes in routine clinical practice and cost implications

**DOI:** 10.1038/bjc.2017.24

**Published:** 2017-02-07

**Authors:** S De Placido, C De Angelis, M Giuliano, C Pizzi, R Ruocco, V Perrone, D Bruzzese, G Tommasielli, M De Laurentiis, S Cammarota, G Arpino, G Arpino

**Affiliations:** 1 Division of Oncology, Department of Clinical Medicine and Surgery, University of Naples Federico II, Naples, Italy; 2 Lester and Sue Smith Breast Center, Dan L. Duncan Comprehensive Cancer Center, Baylor College of Medicine, Houston, TX, USA; 3 Consorzio Nazionale delle Cooperative Mediche, Naples, Italy; 4 National Cancer Institute IRCCS ‘Pascale Foundation', Naples, Italy; 5 LinkHealth srl Heath Economics, Outcomes & Epidemiology, Mugnano di Napoli, Italy

**Keywords:** breast cancer, staging, surveillance, non-metastatic, imaging tests, follow up, PET scan, mammogram, CT scan

## Abstract

**Background:**

Although guidelines do not recommend computerised tomography (CT), positron emission tomography (PET) or magnetic resonance imaging (MRI) for the staging or follow-up of asymptomatic patients with non-metastatic breast cancer, they are often requested in routine clinical practice. The aim of this study was to determine the staging and follow-up patterns, and relative costs in a large population of breast cancer patients living and treated in a Southern Italian region.

**Methods:**

We analysed the clinical computerised information recorded by 567 primary-care physicians assisting about 650 000 inhabitants in the Campania region. Patients with non-metastatic breast cancer were identified and divided into calendar years from 2001 to 2010. The number of diagnostic tests prescribed per 100 patients (N/Pts) and the mean cost per patient was determined 3 months before diagnosis and up to 1 year after diagnosis. Costs are expressed in constant 2011 euros.

**Results:**

We identified 4680 newly diagnosed cases of asymptomatic non-metastatic breast cancer. N/Pts increased significantly (*P*<0.0001) from 2001 to 2010. The mean number of prescribed mammograms, bone scans, abdominal ultrasound and chest X-rays (‘routine tests'), and costs was unchanged. However, the number of CT, PET scans and MRI (‘new tests')prescriptions almost quadrupled and the mean cost per patient related to these procedures significantly increased from €357 in 2001 to €830 in 2010 (*P*<0.0001).

**Conclusions:**

New test prescriptions and relative costs significantly and steadily increased throughout the study period. At present there is no evidence that the delivery of new tests to asymptomatic patients improves breast cancer outcome. Well-designed clinical trials are urgently needed to shed light on the impact of these tests on clinical outcome and overall survival.

The 10-year survival of breast cancer exceeds 70% in most European regions, with an 89% survival for local and 62% for regional disease (Allemani *et al*, 2013). Advances in the early detection and treatment of breast cancer have resulted in lower death rates (American Cancer Society, 2015). The high incidence of breast cancer combined with decreasing mortality rates has led to an increase in the number of breast cancer survivors who need long-term surveillance (Houssami *et al*, 2009). These advances have been paralleled by significant increases in the costs of breast cancer care (Maitino *et al*, 2003). Costs for breast cancer diagnosis/staging and surveillance therefore place a heavy burden on health-care systems and economies (Iglehart, 2006).

Breast cancer staging procedures should be based on a clinical examination combined with bilateral mammography and ultrasound of the breast and regional lymph nodes (Perry *et al*, 2008; Gradishar *et al*, 2016). As asymptomatic distant metastases are very rare, only patients with a large aggressive tumour, or clinical signs, symptoms or laboratory values suggesting metastatic spread at diagnosis should undergo comprehensive laboratory and radiological staging (Harris *et al*, 2007; Khatcheressian *et al*, 2013).

No randomised data exist to support any particular follow-up sequence or protocol in patients with non-metastatic breast cancer. Accurate medical history taking and physical examination, and yearly mammogram are at present the only tools that guarantee early detection of breast cancer recurrence (Dewar and Kerr, 1985; Rosselli Del Turco *et al*, 1994; The GIVIO Investigators, 1994; Montgomery *et al*, 2007; Paszat *et al*, 2009; Khatcheressian *et al*, 2013; Gradishar *et al*, 2016). In asymptomatic patients, there are no data that other laboratory or imaging tests during follow-up produce a survival benefit (Grunfeld *et al*, 1996; Gulliford *et al*, 1997; Rojas *et al*, 2005; Mitchell, 2008; Houssami *et al*, 2009; Dinan *et al*, 2010).

Although there is general agreement about the most appropriate staging and surveillance strategies for patients with non-metastatic breast cancer, (Dewar and Kerr, 1985; Rosselli Del Turco *et al*, 1994; The GIVIO Investigators, 1994; Montgomery *et al*, 2007; Paszat *et al*, 2009; Khatcheressian *et al*, 2013; Gradishar *et al*, 2016) the advent of more sensitive sophisticated imaging tests such as computerised tomography (CT), positron emission tomography (PET) and magnetic resonance imaging (MRI) has profoundly changed imaging test prescriptions in patients with non-metastatic breast cancer in daily clinical practice (Mille *et al*, 2000).

Little is known about current practice patterns of clinicians carrying out breast cancer staging and surveillance in Italy, nor about whether they adhere to or deviate from guideline recommendations (Grunfeld *et al*, 1996, 2006). The aim of this study was to determine the staging and follow-up patterns, and relative costs in a large population of breast cancer patients living and treated in a Southern Italian region. To this aim, we examined the data reported in the clinical charts of primary-care physicians (PCPs) during the last 10 years.

## Materials and methods

### Data source

The data for this analysis were extracted from a primary-care database that contains the charts of ∼650 000 inhabitants living in from the Campania region (Southern Italy) recorded by 567 PCPs. Since 1998, these PCPs have used the same software to record data during their daily practice and receive formal periodic training for data entry. The data underwent a range of quality checks to evaluate the consistency and completeness of information, and each PCP received a validation report after routine data extraction procedures.

An encrypted patient code links demographic details to medical diagnoses, drug prescriptions (drug name, date of prescription, number of packs prescribed), diagnostic procedures and laboratory tests (with their relevant results), hospital admissions and date of death. Vaccinations, allergies, body mass index, blood pressure measurements and some aspects of lifestyle are also recorded. Diagnoses were coded according to the International Classification of Diseases, 9th Revision, Clinical Modification (ICD-9-CM). Drug names were coded according to the Anatomical Therapeutic Chemical (ATC) Classification. The study was approved by the Institutional Review Board of the University of Naples Federico II protocol number 44/10.

### Study population

The study population was constituted by women affected by non-metastatic breast cancer (ICD-9-CM, 174.xx) diagnosed between 1 January 2001 and 31 December 2010. Patients were excluded from the analysis if they died or disenrolled during the 12 months after diagnosis (initial phase) or had other malignancies or metastases before or on the day of the diagnosis of breast cancer. The date of disease onset was defined as the date of the first appearance of the breast cancer code in the PCP's records. We also recorded age, geographic location (rural, semirural or urban) and the Charlson Comorbility Index, calculated as an indicator of general health status at the time of disease onset (Quan *et al*, 2005).

### Diagnostic imaging and cost analysis

We determined the number of imaging tests linked to non-metastatic breast cancer diagnosis. To capture all prescribed tests associated with the initial non-metastatic breast cancer diagnosis, we counted the number of diagnostic imaging tests prescribed 3 months before and 12 months after the date of disease onset for each patient. The imaging examinations linked to breast cancer were grouped into new (i.e., CT, MRI, breast MRI and PET) and routine imaging test (i.e., chest radiograph, abdominal ultrasound, bone scan and mammograms). We evaluated the costs of examinations reimbursed by the Italian NHS based on the Italian NHS tariffs. The Consumer Prices Index (Eurostat) was used to adjust for inflation in cost estimates between 2001 and 2010. We calculated our estimates in terms of 2011 euros because this was the most recent year for which PCP records were available.

### Statistical methods

Descriptive statistics were used to define patients' demographics and comorbid conditions stratified by year of diagnosis. *χ*^2^ statistics for trend and linear-by-linear association were used to compare distributions of categorical and continuous variables, respectively. Statistical significance was defined as a two-sided *P*-value <0.05. The number of routine and new imaging tests per thousand patients by year from diagnosis was calculated and the percentage of patients who received one or more procedures was plotted. For each year of diagnosis, the costs of imaging procedures are expressed in euros as mean cost per patient. Changes in number of procedures and in related cost per patient from 2001 to 2010 are expressed as mean annual rate increases. We estimated the mean annual increases separately for each imaging test using a generalised linear model with a Poisson count distribution and log link for counts, and a log link and normal distribution for costs. All mean annual increase estimates were adjusted for age, geographic location and the Charlson Comorbidity Index. Modelling and statistical analysis were carried out using R version 2.12.1 (IBM SPSS Statistics - Integration Plug-In for R for SPSS Statistics 20 software) and the SPSS software version 17.1 for Windows (SPSS Inc., Chicago, IL, USA).

## Results

### Patients' characteristics

A total of 4680 incident cases of non-metastatic breast cancer recorded from 1 January 2001 to 31 December 2010 were retrieved. The baseline characteristics and the Charlson Comorbidity Index of the study population are reported in Table 1. Neither mean age at diagnosis nor geographical distribution differed significantly in our patients during the study, whereas the Charlson Comorbidity Index was significantly higher in 2001 than in 2010 (0.33 *vs* 0.48; *P*<0.0001).

### Types and costs of imaging tests prescribed from 2001 to 2010

**Figure 1:**
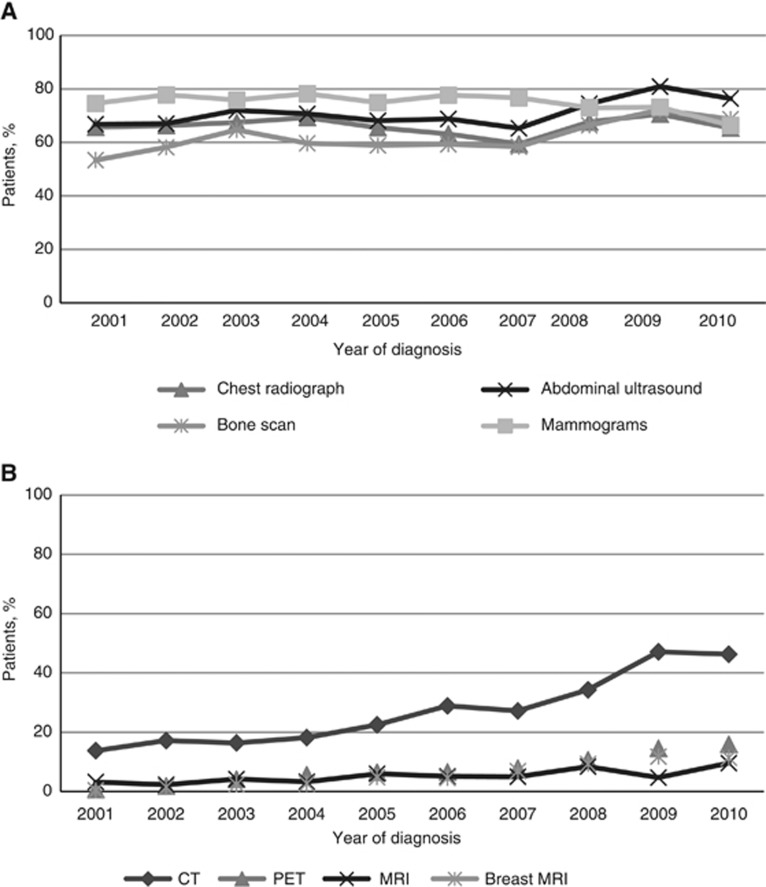
**Trends in the percentage of patients who received one or more routine (**A**) or new (**B**) imaging test(s) in the first year after diagnosis.** CT=computerised tomography; MRI=magnetic resonance imaging; PET=positron emission tomography.

The number and type of tests per patient were recorded 3 months before and 12 months after the date diagnosis of non-metastatic breast cancer from 2001 to 2010. Routine imaging tests, that is, chest radiograph, abdominal ultrasound, bone scan and mammograms, were prescribed more frequently than new imaging tests (Table 2). However, the annual percentage increase in routine imaging test prescriptions was marginal throughout the study (annual increase: 0.1% 95% CI: −0.1 to 0.3). As shown in Figure 1A and Table 2, the annual increase was: −0.8% for chest radiographs (95% CI: −1.8–0.2 to 13.8), 1.9% for abdominal ultrasound (95% CI: 1.0–2.9), 2.2% for bone scan (95% CI: 1.0–3.4) and −0.6% for mammograms (95% CI: −1.8 to 0.5). On the contrary, there was a major change in PET, MRI, breast MRI and CT prescriptions from 2001 to 2010 with an overall annual increase of 15.7% (95% CI: 14.2–17.2). As shown in Figure 1B and Table 2, the annual increase was 11.9% for CT (95% CI: 10.0–13.8), 29.8% for PET (95% CI: 25.0–34.7), 9.0% for MRI (95% CI: 4.9–13.3) and 32.9% for breast MRI (95% CI: 26.3–39.9).

**Figure 2:**
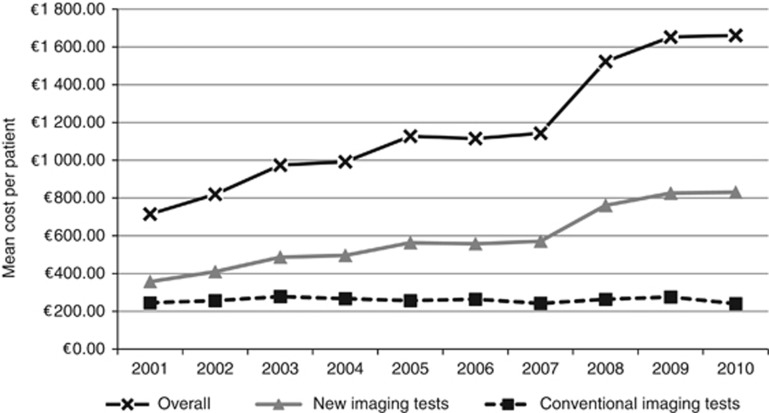
**Trends in mean cost per patient in constant 2011 euros during the first year post diagnosis.Annual increases % and 95% CI estimate were: overall: 10.17 (8.38–12.00).** New imaging tests: 19.39 (15.85–23.04). Routine imaging tests: −0.14 (−0.88 to 0.61).

**Figure 3:**
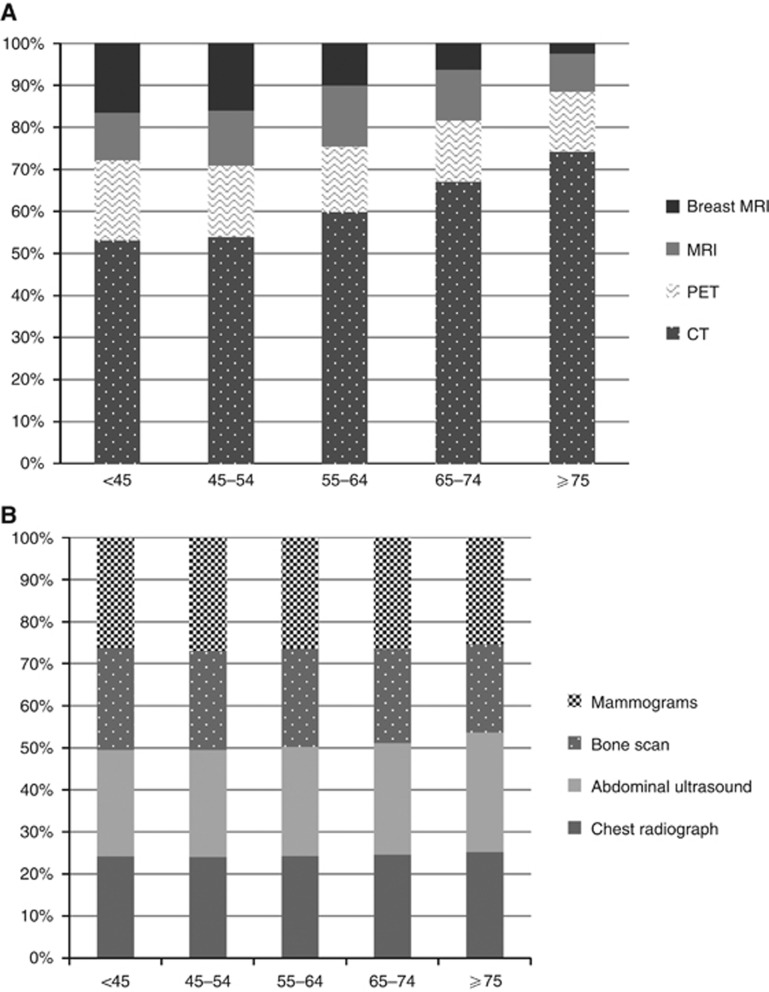
**New (**A**) and routine (**B**) imaging test prescription rates in relation to patients' age.**

The 10-year imaging-related costs increased annually by 10.17% (95% CI: 8.38–12.00). The annual costs of new imaging tests increased significantly each year by 19.39% (95% CI: 15.85–23.04), whereas the annual cost of routine imaging decreased by 0.14% (95% CI: −0.88 to 0.61). The mean cost of imaging tests per patient increased from ∼€650 in 2001 to more than €1600 in 2010 (Figure 2). Importantly, costs for routine imaging tests remained constant (∼€250) and costs for new imaging tests increased from €350 in 2001 to €800 in 2010 (Figure 2). Interestingly, of the new imaging tests, CT scan prescriptions steadily increased and, breast MRI prescriptions decreased in older patients (Figure 3A). There were no age-related differences in routine imaging test prescriptions (Figure 3B).

## Discussion

Recent advances in biomedical imaging have greatly increased the ability of physicians to diagnose and treat a variety of diseases, which, however, is not always associated with a better patient outcome (Mitchell, 2008; Houssami *et al*, 2009; Dinan *et al*, 2010). Technological advances, combined with the practice of defensive medicine, and the patients' demand for more tests, have led to sharp increases in the volume of imaging services and costs.

In this study we analysed the trends of imaging test prescriptions and relative costs in a large population of non-metastatic breast cancer patients diagnosed in a Southern Italian region. Prescription records were extracted from a PCP database over a 10-year period (from 2001 to 2010). Routine imaging test prescriptions and costs remained constant throughout the study with marginal albeit significant annual increases. Chest radiograph and abdominal ultrasound were the most frequently prescribed routine tests throughout the study period and their prescription patterns barely changed over the years. Bone scan prescription rates were also quite high and increased slightly during the study, which indicates that, despite current guidelines (Perry *et al*, 2008; Gradishar *et al*, 2016), most physicians include bone scan in their staging/early follow-up procedures. Not surprisingly, mammograms were also heavily prescribed, and the slight decrease we observed in annual mammogram prescription rates may be due to the increase in the number of breast ultrasounds or breast MRIs. There was no change in routine imaging test prescription patterns in relation to patient age.

New imaging test prescriptions and relative costs significantly and steadily increased during the study. Computerised tomography was the most frequently prescribed new imaging test, particularly for older patients. The most impressive annual increase was for breast MRI and PET scan. Breast MRI is not routinely recommended for non-metastatic patients. However, it may be considered in cases of familial breast cancer associated with BRCA mutations or of large discrepancies between conventional imaging and clinical examination as often occur in young patients (Sardanelli *et al*, 2010). In our study, the largest number of breast MRI prescriptions was for patients below the age of 54 years, as expected given current guidelines (Perry *et al*, 2008; Gradishar *et al*, 2016). The overall annual increase for PET scan prescriptions was about 30%. This rate is very high considering that there is little evidence to support its use in non-metastatic breast cancer patients (Niikura and Ueno, 2010; Robertson *et al*, 2011; Koolen *et al*, 2012).

Age and geographic distribution (rural *vs* urban area) of our patients did not change over the years. Comorbidities were more frequent in patients in the last 4 years than in the earlier years of the study. Therefore, it is unlikely that the increase in comorbidities may have accounted for the increased use of ‘new tests' or related costs we observed. Indeed, a greater availability of more sophisticated imaging techniques and defensive medicine may explain our findings.

A strength of our study is the reliability of the data source. All Italian citizens have equal access to health-care services and are cared for by a general practitioner or PCP within the NHS. Hospital and pharmaceutical services are provided free of charge or at a minimal cost. All cancer patients are eligible for drugs, and imaging and laboratory tests free of charge from the NHS, provided they have a certificate of cancer diagnosis issued by an oncologist working in NHS clinics. Importantly, PCPs participating in this study use a problem-orientated medical record that links prescriptions to diagnostic problems. However, given our lack of information about tumour size and other tumour-related risk factors at diagnosis, and about the reasons that may have led physicians to prescribe more sophisticated and expensive imaging tests, we cannot judge whether the prescriptions were appropriate or not.

In the present study, we analysed only prescriptions from PCPs in Campania, therefore our results may not be representative of the staging and follow-up preferences of Italian PCPs in general or of the Italian oncology community. However, our results coincide with the replies to a recent web-based questionnaire in which 90.4% of Italian oncology units declared they did not apply the minimal breast cancer follow-up procedures after primary treatment in asymptomatic women recommended by national and international oncology societies (Grunfeld *et al*, 2005; Khatcheressian *et al*, 2013; Gradishar *et al*, 2016). Also consistent with our data, a retrospective analysis of the follow-up care of breast cancer patients showed that intensive follow-up testing is a quite common clinical practice in the Italian region of Emilia-Romagna (Leoni *et al*, 2013). Other previous studies also reported that only a few medical oncology units prescribe minimal follow-up procedures (Blamey *et al*, 2007; Barni *et al*, 2011). Another important limitation of our study is that the results may not necessarily apply to other countries because of the differences in terms of financing and organisation of health care, and approaches to pricing and reimbursement. However, it is noteworthy that our finding of an increase in prescriptions of new imaging tests in daily clinical practice is in line with studies conducted in other countries (Dinan *et al*, 2010; Grunfeld *et al*, 2010; Khatcheressian *et al*, 2013; Gradishar *et al*, 2016).

In conclusion, despite a lack of evidence of their effectiveness in large clinical trials, CT, PET and MRI are routinely prescribed for patients affected by non-metastatic breast cancer, and have completely changed diagnostic/surveillance algorithms. There is an urgent need for economic evaluations of breast cancer management to ensure efficient use of health-care resources (Mitchell and Lagalia, 2009). During their medical education, clinicians and trainees should be made aware of the need to avoid overuse, underuse and misuse of scarce medical resources (Cady, 1996; Donnelly *et al*, 2001). Given the massive use of the new imaging tests by physicians in routine daily practice even in asymptomatic patients with non-metastatic breast cancer, and the fact that most data for staging and follow-up recommendations come from an era of less sophisticated diagnostic procedures and less efficacious treatment of advanced disease, well-designed clinical trials are urgently needed to determine the impact of new technologies on clinical outcome and overall survival.

## Figures and Tables

**Table 1:** Baseline characteristics of the study population

	**Year of diagnosis**										
	**2001**	**2002**	**2003**	**2004**	**2005**	**2006**	**2007**	**2008**	**2009**	**2010**	***P*****-value**
*N*	439	418	432	474	529	481	483	499	471	454	
Age											
Mean (±s.d.)	57.78±12.78	61.00±13.12	59.00±13.34	59.51±13.31	59.53±12.75	59.20±13.53	59.51±12.91	59.22±13.87	59.55±13.96	59.72±13.96	0.146
Charlson index											
Mean (±s.d.)	0.33±0.48	0.29±0.47	0.32±0.49	0.29±0.48	0.32±0.48	0.38±0.52	0.37±0.52	0.39±0.54	0.41±0.55	0.48±0.58	<0.0001
Location, *n(%)*											
Urban	353 (90.1%)	346 (88.3%)	350 (86.8%)	383 (88.0%)	727 (88.4%)	382 (86.8%)	407 (91.5%)	413 (90.8%)	381 (90.3%)	350 (86.8%)	
Semirural	33 (8.4%)	44 (11.2%)	50 (12.4%)	51 (11.7%)	49 (10.1%)	55 (12.5%)	35 (7.9%)	37 (8.1%)	35 (8.3%)	50 (12.4%)	
Rural	6 (1.5%)	2 (0.5%)	3 (0.7%)	1 (0.2%)	7 (1.4%)	3 (0.7%)	3 (0.7%)	5 (1.1%)	6 (1.4%)	3 (0.7%)	

**Table 2:** Imaging test prescriptions per year

	**Year of diagnosis (No of tests per 100 patients)**										
**Imaging test**	**2001**	**2002**	**2003**	**2004**	**2005**	**2006**	**2007**	**2008**	**2009**	**2010**	**Annual increase % (95% CI)**
**Routine**											
Chest radiograph	100.5	112.2	122.0	114.1	110.4	110.2	94.6	107.4	117.2	95.2	−0.8 (−1.8 to 0.2)
Abdominal ultrasound	117.1	120.3	140.5	134.6	127.2	135.8	125.1	142.9	152.9	135.7	1.9 (1.0 to 2.9)
Bone scan	73.1	76.3	84.5	80.2	80.0	82.7	77.4	88.6	96.0	90.1	2.2 (1.0 to 3.4)
Mammograms	87.9	90.2	89.1	91.4	86.8	90.4	88.6	88.2	87.7	77.5	−0.6 (−1.8 to 0.5)
Total	378.6	399.0	436.1	420.3	404.3	419.1	385.7	427.1	453.7	398.5	0.1 (−0.1 to 0.3)
**New**											
CT	23.9	34.4	35.0	33.5	39.1	45.5	39.8	55.5	72.8	74.7	11.9 (10.0 to 13.8)
PET	0.7	2.4	4.6	7.0	9.3	7.9	11.4	17.4	22.3	22.2	29.8 (25.0 to 34.7)
MRI	5.9	4.1	7.2	6.1	9.5	6.9	9.5	12.6	7.4	14.1	9.0 (4.9 to 13.3)
Breast MRI	0.5	1.0	2.5	3.0	5.7	4.8	7.2	10.8	13.0	13.2	32.9 (26.3 to 39.9)
Total	32.3	43.5	49.8	50.4	65.0	68.2	69.6	101.4	120.0	128.9	15.7 (14.2 to 17.2)

Abbreviations: CT=computerised tomography; MRI=magnetic resonance imaging; PET=positron emission tomography.
